# Supplementary material for: Splice-Junction-Based Mapping of Alternative Isoforms in the Human Proteome
Source: Cell Rep. Author manuscript; Available in PMC 2020 Jan 15. (PMC6961840; doi:10.1016/j.celrep.2019.11.026)
Supplement: 3 [file NIHMS1546469-supplement-3.zip › DF2/PXD009021/Liver-85-P02760-TVGDEELLR.pdf]

A

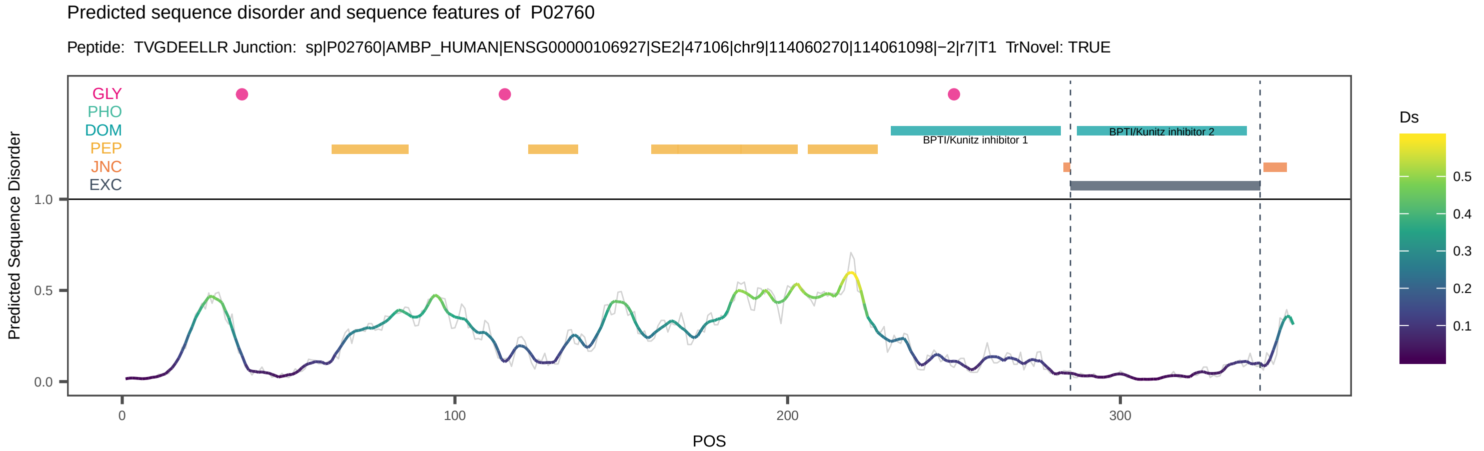

Predicted sequence disorder and sequence features of P02760
Peptide: TVGDEELLR Junction: sp|P02760|AMBP_HUMAN|ENSG00000106927|SE2|47106|chr9|114060270|114061098|−2|r7|T1 TrNovel: TRUE
GLY
PHO
DOM
PEP
JNC
EXC
BPTI/Kunitz inhibitor 1
BPTI/Kunitz inhibitor 2
Predicted Sequence Disorder
1.0
0.5
0.0
0
100
200
300
POS
Ds
0.5
0.4
0.3
0.2
0.1

B

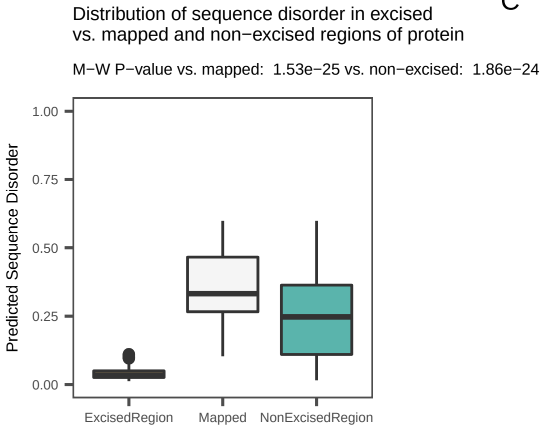

Distribution of sequence disorder in excised
vs. mapped and non−excised regions of protein
M−W P−value vs. mapped: 1.53e−25 vs. non−excised: 1.86e−24
Predicted Sequence Disorder
1.00
0.75
0.50
0.25
0.00
ExcisedRegion
Mapped
NonExcisedRegion

C
